# Hyperbaric oxygen therapy: a possible choice for patients with resistant thin endometrium during frozen embryo transfer treatments

**DOI:** 10.1186/s12958-023-01123-4

**Published:** 2023-09-01

**Authors:** Jingjing Chen, Fangling Huang, Jing Fu, Jianjuan Zhao, Jinsheng Li, Zhengrong Peng, Jing Zhao, Bin Xu, Shuyi Li, Qiong Zhang, Shaolin Liang, Yanping Li

**Affiliations:** 1 Department of Reproductive Medicine, Xiangya Hospital, Central South University, 87 Xiangya Road, Changsha, Hunan Province 410008 China; 2 Clinical Research Center for Women’s Reproductive Health in Hunan Province, Changsha, Hunan Province 410008 China; 3 Department of Hyperbaric Oxygen, Xiangya Hospital, Central South University, Changsha, Hunan Province 410008 China; 4 STI-Zhilian Research Institute for Innovation and Digital Health, #1203, Building 1, No. 21, 18 Fuxing Road, Haidian District, Beijing, 410000 China; 5 Department of Hyperbaric Oxygen, The First Affiliated Hospital, Southern University of Science and Technology, Shenzhen, 518071 China; 6 Institute for Six-sector Economy, Fudan University, Shanghai, 200433 China; 7 Xiangya Hospital, “Mobile Health” Ministry of Education-China Mobile Joint Laboratory, Central South University, Changsha, Hunan Province 410008 China

**Keywords:** Resistant thin endometrium, Hyperbaric oxygen therapy, Frozen embryo transfer, Asherman syndrome

## Abstract

**Background:**

Thin endometrium is considered suboptimal for embryo implantation, leading to compromised pregnancy rates without effective therapies. While some studies have reported promoted endometrial growth after a period of hyperbaric oxygen therapy (HBOT) in patients with intrauterine adhesion, there have been no reports in patients with resistant thin endometrium. The purpose of this study was to investigate the impact of HBOT on endometrium growth and pregnancy outcomes in patients with resistant thin endometrium during frozen embryo transfer (FET) treatments.

**Methods:**

This prospective pre-post cohort study was conducted at a university-affiliated assisted reproductive medical center between October 2021 and December 2022. Patients who had experienced at least one canceled transfer cycle due to a thin endometrium(< 7 mm) on the endometrium transformation day, despite the use of standard therapies as well as adjuvant therapies, were enrolled in the study. Patients were assigned voluntarily to either the HBOT group or the concurrent control group. The HBOT group received daily HBOT for at least 10 days during the proliferative phase, in addition to the routine endometrium preparation methods and the concurrent control group underwent cycles without HBOT. Propensity score matching (PSM) was used to ensure comparability between the groups. Both self-control and case-control comparisons were conducted. The primary outcome measured was endometrial thickness (ET) on the day of endometrium transformation. Secondary outcomes included intrauterine pregnancy rate (IPR), embryo implantation rate (IR), miscarriage rate, and others.

**Results:**

Patients in the HBOT group demonstrated a significantly thicker endometrial thickness on the day of endometrium transformation after undergoing therapy (5.76 ± 1.66 vs. 6.57 ± 1.23, P = 0.002). This improvement was accompanied by a decreased rate of cycle cancellations. Baseline parameters and endometrial thickness were comparable between the HBOT group and the concurrent control group during the cycle. The IPR was similar in patients who received cleavage-stage embryos (0.0% vs. 6.7%, P = 1.00), but significantly higher in patients in the HBOT group who received blastocysts (53.8% vs. 18.2%, P = 0.017).

**Conclusions:**

A period of HBOT prior to endometrium transformation contributes to increased endometrial thickness and facilitates blastocyst implantation in patients with resistant thin endometrium during FET treatments.

**Trial registration:**

The trial was registered on the Chinese Clinical Trial Registry (registration no. ChiCTR2300072831, retrospectively registered).

## Background

Endometrial thickness is one of the standard parameters that is used to evaluate endometrial receptivity and predict pregnancy outcomes. While pregnancies have been reported to be possible at an endometrial thickness of 4 mm [1], a thin endometrium is often considered suboptimal for embryo implantation and is associated with compromising implantation, pregnancy, and live birth rates [2, 3]. However, the threshold that defines “thin endometrium” remains a subject of controversy [4–6].

Treatment for thin endometrium is an ongoing challenge. Different approaches have been proposed to improve refractory endometrium, including extended or alternative routes for estrogen support [7, 8], adjuvant aspirin, sildenafil [9, 10], intrauterine perfusion granulocyte colony-stimulating factor (G-CSF) [11, 12] and autologous platelet-rich plasma [13]. More recently, stem cell therapy has also been considered a promising alternative [14]; however, to date, most of the studies are preliminary, and there are significant challenges regarding safety, high costs, and effectiveness [15]. Despite the use of these therapies, a subset of patients continues to exhibit unresponsiveness within their endometrium, consequently encountering repeatedly canceled transfer cycles or implantation failure. Therefore, new therapeutic approaches for thin endometrium are needed.

Hyperbaric oxygen therapy (HBOT) refers to a therapeutic method in which a patient inhales 100% pure oxygen at an elevated atmospheric pressure inside a highly pressured chamber [16]. It has been considered a promising treatment as either a primary or alternative therapy for the management of some complex diseases, including chronic wounds, injuries, ischemic diseases, infections, etc., over the past three decades [17–19]. To date, a few studies have reported the application of HBOT in infertility patient groups, either in patients with impaired endometrium or poor prognosis [20–23]. After a period of HBOT, observations of increased endometrial thickness and improved endometrial quality were reported, along with a live birth in a patient with scarred endometrium and five failed transfer cycles. Despite the promising results, most of the studies were case or case series, and had a mixed effect of HBOT on both the endometrium and oocytes. Therefore, this study was designed aiming to investigate the effect of HBOT on the endometrium, particularly in patients with thin endometrium who are unresponsive to conventional treatments, during frozen embryo transfer (FET) treatments.

## Materials and methods

### Study population

This is a prospective pre-post cohort study conducted at the Department of Reproductive Medicine of Xiangya Hospital, Central South University, between October 2021 and December 2022. The study protocol was approved by the Reproductive Medicine Ethics Committee of Xiangya Hospital (reference number: 2,021,008) and registered on the Chinese Clinical Trial Registry (registration no. ChiCTR2300072831). Written consent was obtained from all the patients. The inclusion criteria were as follows: (i) patients undergoing frozen embryo transfer procedures; (ii) had at least one canceled transfer cycle due to a resistant thin endometrium (< 7 mm) on the endometrium transformation day, despite the use of estradiol valerate combined with other adjuvant therapies, including aspirin, G-CSF, vaginal sildenafil, etc. Patients were excluded for the following reasons: (i) endometrial polyps, tuberculosis, dysplasia, or cancer; (ii) uterine malformations; and (iii) accompanied with other diseases, including diabetes and lupus erythematosus.; (iv) medical contraindications to HBO therapy, including ear injury and thoracic surgery, uncontrolled hypothermia, claustrophobia, etc.; (v) did not commence FET within 3 months after HBOT; and (vi) did not want to participate in the study.

### Study groups and propensity score matching

Patients who met the inclusion criteria were suggested to HBOT before commencing the next transfer cycle, and whether they received the treatment or not was decided jointly by both the patient and the physician. Those who received HBOT were assigned to the HBOT group and the others were assigned to the concurrent control group. Propensity score matching (PSM) was used to avoid the potential bias between the groups, and patients were matched in a 1:1 ratio for age, body mass index (BMI), and endometrial thickness on the endometrium transformation day before HBOT. Notably, for patients with intrauterine adhesions, the American Fertility Society’s (AFS) classification of intrauterine adhesions was used and matched between the groups. In addition, to exclude a possible effect of accumulated embryo transfer on pregnancy outcomes, the comparisons were made on the same transfer cycle of a pair of patients. After matching, 41 pairs of patients were selected for statistical comparison from a total of 146.

### HBO therapy

Considering the potential value of HBOT in supporting endometrium growth and evaluating the detrimental effect of high oxygen tensions on embryo development [24], we considered that the therapy should be carried out for at least 10 days during the proliferative phase in addition to routine endometrium preparation methods and ceased on the endometrium transformation day, in accordance with previous studies [20, 22].

For patients who were willing to take the therapy, HBOT was given once a day at the Department of Hyperbaric Oxygen, Xiangya Hospital, Central South University. The treatments were performed in a multiplace hyperbaric chamber pressurized with air (YC2410-24, Hoto Oxygen Industrial, Shandong, China) at a target pressure of 2.5 atm absolute (ATA) for 100 min total treatment time (increased pressure for 20 min, ordinary pressure for 60 min, and decreased pressure for 20 min). Five minutes after the start of the pressurization, patients breathed 100% isobaric oxygen for 70 min and had a 5-minute break during ordinary pressure to minimize the side effects of the treatment. During the therapy, patients were closely monitored by the physicians in case of adverse reactions.

### Endometrium preparation and embryo transfer

Vitrified-warmed embryos were transferred to all patients in the study. Different endometrium preparation methods, including hormone replacement treatments (HRT), ovulation monitoring (natural cycles, NC), and GnRH-agonist combined hormone replacement treatments (GnRH-a HRT), were employed based on the evaluation of a patient.

The criteria for embryos of good-quality were as follows: (i) cleavage-stage embryo: ≥7 blastomeres and < 20% fragmentation on day 3 [25, 26]; (ii) blastocysts: ≥3BB on day 5 or day 6 [27].

### Ultrasound measurement

Endometrial features, including endometrial thickness and endometrial pattern, were recorded during the cycle by transvaginal 8 MHz ultrasonography with Doppler ultrasound (GE Voluson S6, GE Medical Systems, USA). The endometrial thickness (ET) was measured on a median longitudinal plane of the uterus to measure the maximum distance between the endometrial-myometrial surface from the anterior to the posterior wall of the uterus. Thin endometrium was defined as ET < 7 mm [28], either on the ovulation day during NC cycles despite a normal serum estradiol level or on the day of progesterone administration even after the maximum dose of oral estradiol valerate was administered for over 12 days during HRT or GnRH-a HRT cycles. The endometrial pattern was classified as pattern A (a triple-line pattern), B (an intermediate isoechogenic pattern), or C (a homogenous hyperechogenic pattern).

### Follow-up and data collection

Patient follow-up was performed by telephone consultation until March 2023. Any complications with the HBOT were self-reported and recorded.

The primary outcome of the study was the endometrial thickness on the endometrium transformation day. The secondary outcomes included the embryo implantation rate (IR), intrauterine pregnancy rate (IPR), biochemical pregnancy rate (BPR), miscarriage rate, etc.

### Statistical methods

Statistical analysis was carried out by SPSS Statistics version 25.0 (IBM, USA). Continuous data were expressed as the mean and standard deviation or median and range, depending on the distribution. Paired Student’s t test, Fisher’s exact test, Mann-Whitney U test, and chi-square test were used when appropriate. A two-sided P < 0.05 was considered statistically significant.

## Results

### Participants

**Fig. 1:**
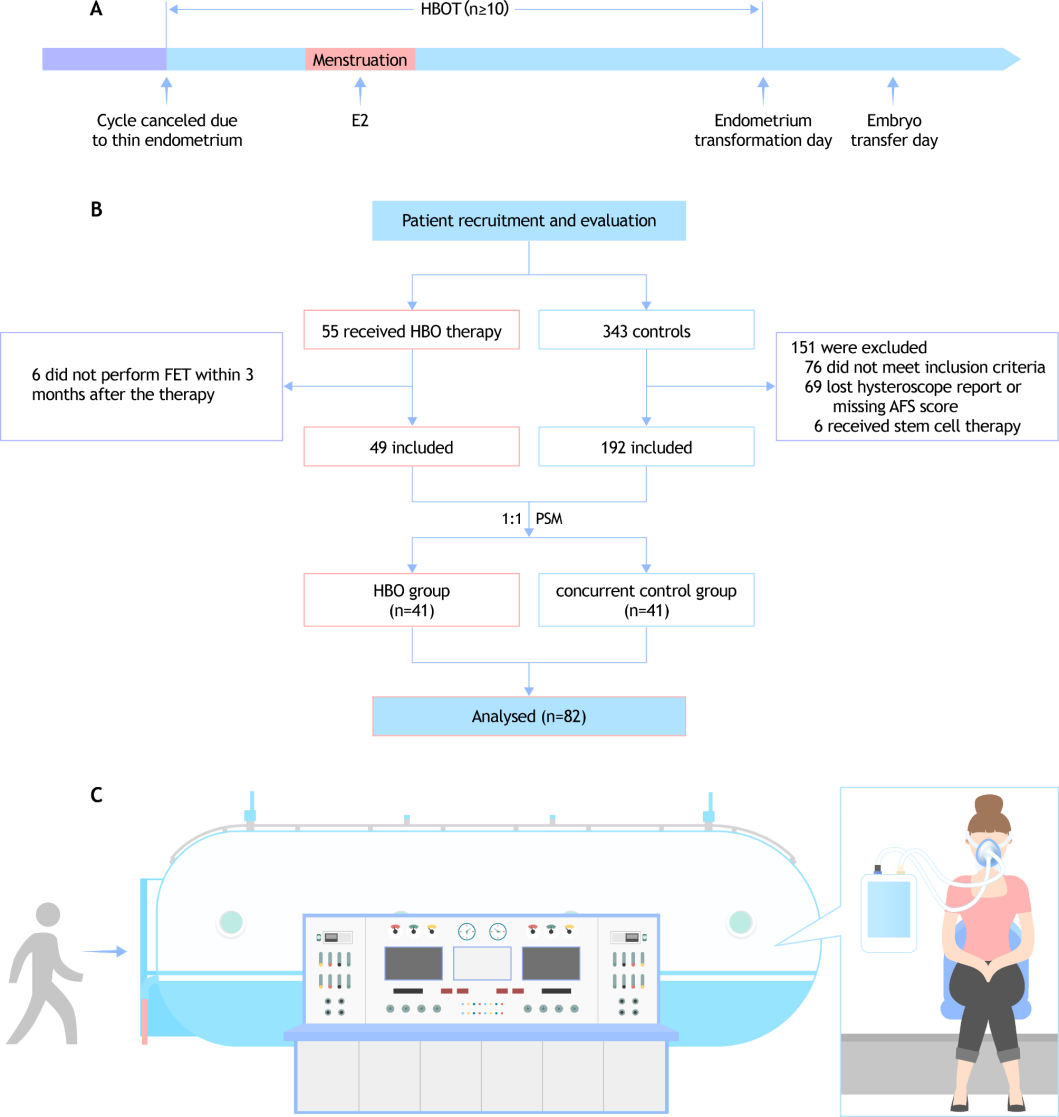
Study design and flowchart of the patient enrollment. **(A)** Flowchart showing the study design. **(B)** Flowchart of the patient enrollment. **(C)** Sketch of the hyperbaric oxygen therapy. HBOT, hyperbaric oxygen therapy; E2, estradiol; FET, frozen embryo transfer; AFS, the American Fertility Society’s (AFS) classification of intrauterine adhesions; PSM, propensity score matching

Between October 2021 and December 2022, a total of 398 patients with thin endometrium underwent frozen embryo transfer procedures in our center. After preliminary evaluation for meeting the described inclusion and exclusion criteria and after counseling, 55 consented to HBOT and were enrolled in the study. The rest were assigned to the concurrent control group. After PSM, 41 paired patients were included for analysis (Fig. 1). The patients reported no complications, such as headache or discomfort, during the study.

### Improvement in endometrial thickness after HBOT

**Fig. 2:**
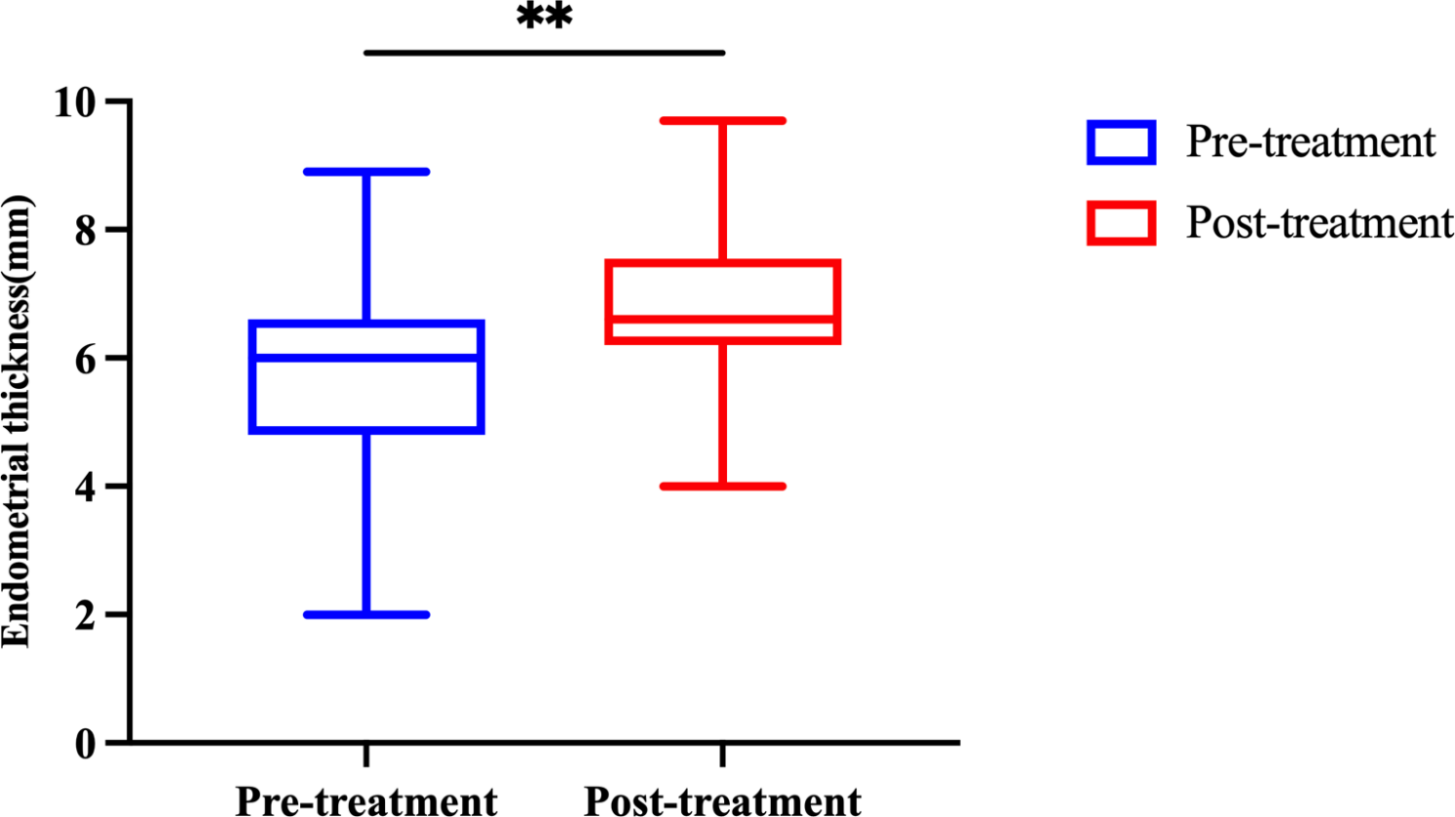
Improvement of endometrial thickness before and after the HBOT. Results are shown as the mean ± standard deviation, **P < 0.01

To evaluate the effects of HBOT on endometrial receptivity, pre- and post-therapy endometrial parameters were compared, as shown in Table 1 and Fig. 2. Before HBOT, a total of 76 transfer cycles were conducted, 47 (61.8%) of which were canceled due to the appearance of uterine cavity fluid or resistant thin endometrium on the planned endometrium transformation day. After the therapy, a significant decrease in the cycle cancellation rate was observed (11, 19.0%), and more patients were considered eligible for embryo transfer. No significant differences were found in the initial ET; however, after the therapy, the ET on the endometrium transformation day was significantly thicker (5.76 ± 1.66 vs. 6.57 ± 1.23, P = 0.002), despite estrogen being administered for a longer duration before (15.00 [13.00, 15.00] vs. 13.00 [12.00, 15.00], P = 0.057). Patients showed similar endometrial patterns before or after the therapy. No statistically significant difference was found in the change in ET from the endometrium transformation day to the embryo transfer day (-0.20 ± 1.62 vs. -0.16 ± 0.96, P = 0.922).

**Table 1:** Basic and clinical characteristics before and after HBOT

	Before HBOT	After HBOT	P-value
Total Cycles, n	76	58	-
Cycle Cancellation Rate, n (%)	47(61.8)	11(19.0)	0.001
Endometrial Preparation Method, n (%)			0.042
NC-FET	23 (30.3)	12 (20.7)	
HRT-FET	33 (43.4)	38 (65.5)	
GnRH-a FET	16 (21.1)	8 (13.8)	
IVF-ET	4 (5.3)	0	
Initial ET, mm	4.46 ± 1.21	4.14 ± 1.17	0.174
ET on Endometrial Transformation Day, mm	5.76 ± 1.66	6.57 ± 1.23	0.002
Duration of Estrogen Administration, d	15.00 [13.00, 15.00]	13.00 [12.00, 15.00]	0.057
Endometrial Pattern on Endometrial Transformation Day^a^, n (%)			0.710
A	3 (3.9)	1 (1.7)	
B	47 (61.8)	35 (60.3)	
C	26 (34.2)	22 (37.9)	
P levels on Endometrial Transformation Day, ng/ml	0.21 ± 0.17	0.28 ± 0.36	0.426
ET on Embryo Transfer Day, mm	7.06 ± 1.99	6.61 ± 1.32	0.429
Change of ET from Endometrium Transformation Day to Embryo Transfer Day, mm	− 0.20 ± 1.62	-0.16 ± 0.96	0.922

^a^Endometrial pattern was classified as A (a triple-line pattern), B (an intermediate isoechogenic pattern) and pattern C (homogenous hyperechogenic pattern)

NC, natural cycle; HRT, hormone replacement cycle; FET, frozen embryo transfer; IVF-ET, in vitro fertilization embryo transfer; ET, endometrium thickness; P, progesterone

### Improvement in pregnancy outcomes

A total of 41 pairs of patients were included and analyzed, as shown in Table 2. The cycle cancellation rate was similar in both groups (14.6% vs. 9.8%, P = 0.737). The baseline parameters, including age, BMI, baseline follicular stimulating hormone (FSH), luteinizing hormone (LH), anti-Müllerian hormone (AMH), infertility duration, etc., were comparable. The groups had a similar proportion of intrauterine adhesion (IUA) patients, and those in the HBOT group had comparatively higher AFS scores before hysteroscopic adhesiolysis. Patients in the HBOT group received on average 28.48 ± 15.24 HBO treatments. No statistically significant difference was found in ET during the cycle between the groups; however, estrogen was administered in the concurrent control group for a longer duration (12.50 [12.00, 15.00] vs. 16.00 [14.50, 17.00], P < 0.001). Patients in the concurrent control group received more embryos and embryos of good quality; however, the embryo implantation rate was significantly higher in the HBOT group (33.3% vs. 11.1%, P = 0.008). A subgroup analysis was performed based on the stage of the transferred embryos. The IPR and the BPR were similar in patients who received cleavage stage embryos (0.0% vs. 6.7%, P = 1.00; 11.1% vs. 20.0%, P = 1.00), while in patients who received blastocysts, the IPR was significantly higher in the HBOT group (53.8% vs. 18.2%, P = 0.017).

**Table 2:** Baseline parameters and clinical outcomes of the paired patients

	HBOT	Concurrent Control	p-value
Patients, n	41	41	
Cycle Cancellation Rate, n (%)	6 (14.6)	4 (9.8)	0.737
Age, y	35.32 ± 4.98	35.24 ± 4.03	0.942
BMI, kg/m^2^	23.09 ± 6.99	22.00 ± 2.53	0.348
Baseline FSH, mIU/ml	7.03 ± 3.45	6.26 ± 3.18	0.351
Baseline LH, mIU/ml	6.02 ± 3.60	6.02 ± 5.04	0.995
AMH, ng/ml	3.22 ± 2.77	3.48 ± 2.81	0.678
Infertility Duration, y	3.80 [1.50, 6.50]	4.00 [2.11, 7.00]	0.422
Patients with a History of IUA, n (%)	26 (63.4)	25 (61.0)	0.820
AFS Score	7.54 ± 2.61	6.50 ± 3.41	0.212
Infertility type, n (%)			
Primary Infertility	5 (12.2)	9 (22.0)	0.379
Secondary Infertility	36 (87.8)	32 (78.1)	
Etiology of Infertility, n (%)			
Tubal	20 (48.8)	20 (48.8)	0.343
DOR	3 (7.3)	3 (7.3)	
Male factors	2 (4.9)	2 (4.9)	
RSA/PGT	14 (34.2)	8 (19.5)	
IUA	2 (4.9)	6 (14.6)	
Others	0 (0)	2 (4.9)	
Average HBOT treatments, n	28.48 ± 15.24	-	-
Endometrial Preparation Method, n (%)			0.316
NC-FET	8 (19.5)	14 (34.1)	
HRT-FET	26 (63.4)	22 (53.7)	
GnRH-a FET	7 (17.1)	5 (12.2)	
Initial ET, mm	4.20 ± 1.24	4.48 ± 1.07	0.356
Duration of Estrogen Administration, day	12.50 [12.00, 15.00]	16.00 [14.50, 17.00]	< 0.001
ET on Endometrium Transformation Day, mm	6.70 ± 1.21	6.59 ± 1.57	0.712
P levels on Day of Progesterone Administration, ng/ml	0.30 ± 0.41	0.32 ± 0.38	0.893
Endometrial Pattern on Endometrium Transformation Day ^a^, n (%)			
A	1 (2.4)	2 (4.9)	0.392
B	26 (63.4)	20 (48.8)	
C	14 (34.2)	19 (46.3)	
ET on Embryo Transfer Day, mm	6.75 ± 1.26	6.05 ± 1.06	0.456
Num. of Embryo Transferred	1.20 ± 0.41	1.46 ± 0.51	0.019
Num. of Good-Quality Embryos Transferred	0.69 ± 0.68	1.03 ± 0.76	0.049
Embryo Implantation Rate, n (%)	14 (33.3)	6 (11.1)	0.008
Num. of Patients Received D3 Embryos, n	9	15	
Intrauterine Pregnancy Rate, n (%)	0 (0)	1 (6.7)	1
Biochemical Pregnancy Rate, n (%)	1 (11.1)	3 (20.0)	1
Miscarriage, n (%)	-	0 (0)	-
Num. of Patients Received D5 Embryos	26	22	
Intrauterine Pregnancy Rate, n (%)	14 (53.8)	4 (18.2)	0.017
Biochemical Pregnancy Rate, n (%)	2 (7.7)	3 (13.6)	0.649
Miscarriage, n (%)	0 (0)	1 (25)	0.222

^a^Endometrial pattern was classified as A (a triple-line pattern), B (an intermediate isoechogenic pattern) and pattern C (homogenous hyperechogenic pattern)

IUA, intrauterine adhesion; AFS, the American Fertility Society’s (AFS) classification of intrauterine adhesions; NC, natural cycle; HRT, hormone replacement cycle; FET, frozen embryo transfer; IVF-ET, in vitro fertilization embryo transfer; ET, endometrium thickness; P, progesterone

## Discussion

This study presents, to our knowledge, the first and largest cohort of patients with resistant thin endometrium receiving HBO therapy during frozen embryo transfer procedures. Most of the patients were diagnosed with IUA and found to be resistant to common adjuvant therapies, including aspirin, herbal medicine, vaginal sildenafil, etc. After HBO treatment, a significant increase in endometrium thickness on the endometrial transformation day was observed, followed by a decreased cycle cancellation rate. Despite the improvement in the endometrium, the ET and the endometrial pattern were comparable during the cycle between the HBOT and the concurrent control group. However, the IR and the IPR were significantly higher in patients in the HBOT group who received blastocysts.

Endometrial thickness monitoring in relation to the standard cycle is a crucial step during fertility treatment, and it can be easily measured by transvaginal ultrasound [29]. The relationship between thin endometrial thickness and negative pregnancy outcomes has been well documented, although it is a controversial topic [30]. The underlying mechanism of suboptimal endometrial development could be complicated. Common causes of thin endometrium are iatrogenic, e.g., Asherman syndrome, infection, or the use of antiestrogenic drugs. However, it could also be inherently present or occur for reasons that are less understood, e.g., high blood flow impedance of the uterine arteries and subsequently reduced endometrial blood flow [28, 31, 32]. In this study, we included patients with a thin endometrium (< 7 mm) who showed refractory responses to extended estradiol support or other adjuvant therapies, including aspirin and G-SCF in the previous cycles. Most of them were diagnosed with Asherman syndrome under hysteroscopic observation and underwent rounds of hysteroscopic adhesiolysis to restore the uterine cavity; however, it seems that simple removal of the blockage is insufficient. In addition, the reason for the resistant thin endometrium in the rest of the patients remained unclear.

Upon reviewing our data, for patients after HBOT, the ET was significantly thicker on the endometrium transformation day even when estrogen was administered for a relatively shorter period, indicating a good tolerance of the endometrium to hyperoxia and an improved response to exogenous estrogen, inconsistent with LEVERMENT et al. [23] and MITROVIC et al. [22]. However, no significant change was found in the endometrial pattern after therapy.

The improvement in endometrial receptivity could be reflected in pregnancy outcomes. Although pregnancies have also been reported at an endometrial thickness of 4 mm [33], the clinical pregnancy rate increases greatly with increased endometrial thickness [6]. In our study, the cycle cancellation rate decreased significantly after the HBOT (61.8% vs. 19.0%, P = 0.001), and more patients were considered eligible for embryo transfer. When compared to the matched concurrent controls group, the HBOT group received estrogen for a significantly shorter period to reach a similar ET. A subgroup analysis was performed based on the stage of the transferred embryos. The IPR was similar in patients who received day-3 cleavage stage embryos (0.0% vs. 6.7%, P = 1) but significantly higher in the HBOT group in patients who received day-5 or day-6 blastocysts (53.8% vs. 18.2%, P = 0.017). The difference in IPR between the subgroups could potentially be attributed to various factors. One possible explanation could be the limited sample size, with only 9 and 15 patients in each group receiving cleavage stage embryos, which might have led to the possible insignificance of the HBOT effect. Additionally, it is essential to note that despite the observed improvements after the therapy, the endometrial thickness remained relatively thin. In such cases, blastocysts may exhibit greater competence in overcoming the limitations of the thin endometrium. The IPR reported in patients with an endometrium < 7 mm ranged from 7.0–55.7% [3, 6, 11, 34, 35] while noticing the differences in sample size and that a subgroup analysis based on the embryo stage was lacking in some of the studies. We postulate that the higher pregnancy rate could partly be attributed to the increased ET, and also an improvement in endometrial receptivity; however, more endometrial biological indicators, including endometrial vascularization, uterine and subendometrial blood flow, endometrial volume, and histological analysis, should be carried out to verify the treatment effect of HBOT in refractory thin endometrium.

The impact of a thin endometrium on embryo implantation is complicated. The endometrium is composed of two layers: the relatively intact basal layer and the functional layer, which undergoes dynamic changes throughout the menstrual cycle; it is metabolically active and requires appropriate uterine blood flow. After ovulation, the spiral arteries constrict and diminish blood flow to the outer functional layer of a capillary network, creating a low-oxygen environment, which is a prerequisite for embryogenesis and fetal development[24, 36]. Additionally, a thinner functional layer may subject the embryos to higher vascularity and oxygen concentrations from the basal endometrium [37]. Also, a thin endometrium is characterized by high uterine blood flow impedance and poor vascular development, which further results in poor endometrial growth [32].

Physiologically, only a limited amount of oxygen is dissolved in the blood under normal atmospheric pressure, and 80% of the molecular oxygen is utilized by mitochondria [38]. During the menstrual cycle, especially proliferative phases, the epithelial cells have to maintain a necessary degree of ribosomal and mitotic activity as well as glycogen synthesis, which is dependent on aerobic oxidation and is indispensable for embryo implantation. Poor perfusion, along with a thin endometrium and an insufficient mitochondrial supply of oxygen, results in inadequate energy and higher levels of free oxygen radicals, which are detrimental to embryo implantation. The application of mild hyperbaric oxygen offers sufficient oxygen in the plasma to meet the high demand for proliferative endometrium. It can especially restore normoxia in patients with scarred or thin endometrium [21], which supports endometrium growth while avoiding excessive production of reactive oxygen free radicals [19, 21]. However, considering the negative effect of high oxygen concentrations on embryo implantation [24], the time to cease the treatment is important. Patients in the study received a relatively long period of HBOT and stopped the treatment on the endometrial transformation day. Therefore, we believe that the endometrium benefits most from improved perfusion and estrogen support and becomes relatively hypoxic after therapy ceases, which coordinates with physiological transformation and facilitates embryo implantation.

Indications for HBOT include a wide range of diseases, including ischemia-reperfusion injury, intoxication, and central nervous system injury, along with pharmacological effects on poorly perfused tissues resulting from burn injuries, diabetes, chronic wounds, etc., through the creation of oxygen tension gradients that stimulate angiogenesis, perfusion, and vascularization [39–42]. However, the underlying mechanism of HBOT is complicated, and a possible effect of HBOT on subendometrium vasculature remains to be investigated. Impaired angiogenesis and low uterine blood flow were characterized in patients with thin endometrium, and an improvement in endometrial thickness and radial artery resistance index (RI) was observed after treatment with Vitamin E aiming to improve capillary blood flow [26, 32]. Similarly, a study in patients with unexplained infertility observed intensive neoangiogenic processes of the endometrium with low resistance (RI < 0.45) in cycles covered with HBOT at 2.3 ATA [22]. In our study, no significant difference was found in endometrial pattern after the HBOT, and further studies should be carried out to verify the change in endometrial blood flow.

Another possible effect of HBOT could be the synergistic effect with exogenous drugs. It was reported to promote the delivery and efficacy of antitumor drugs in solid tumors by ameliorating the microenvironment and disrupting hypoxia-mediated immunosuppression [43, 44]. In our study, patients in the HBOT group received exogenous estrogen for significantly shorter periods to reach a similar ET as the concurrent controls group, which reflects promoted responses in the HBOT Group. However, for ethical reasons, adjuvant therapies, including G-CSF intrauterine perfusion, aspirin, etc., were also used in addition to HBOT in some of the patients according to practical circumstances. We failed to find a significant effect of the combined therapies, which was probably due to the limited sample size. Well-designed clinical trials are needed for further evaluation of the effect of these combined therapies.

In our study, no adverse reactions were reported during the therapy. In contrast, two patients found improvements in sleep quality after the treatment, and one patient observed vision improvements. HBOT was reported to improve fatigue, sleep disturbance, and quality of life in patients with fibromyalgia [45], post-COVID-19 syndrome [46], or cancer survivors with pelvic radiation injuries [47]. While acknowledging that both infertility diagnosis and treatment result in significant psychological distress [48], we postulate a possible effect of HBOT on both the mental and physical health of an infertile patient; however, well-designed studies are needed before a conclusion.

As an adjunctive therapy for patients with thin endometrium, HBOT has advantages in multiple aspects, including its noninvasive nature, convenience, and potential value in systemic improvement. However, the therapy requires good medical compliance from a patient for a relatively long period of time and requires a pressurized chamber as well as close monitoring by healthcare providers.

Nonetheless, the study had several limitations. First, the sample size was limited, and patients received HBOT at their own will and were not randomly assigned. Second, the duration of HBOT as well as the method of preparing endometrium were not strictly controlled but decided based on the time of a patient’s visit and her physical condition. Third, we were unable to record uterine and subendometrial blood flow as well as uterine artery hemodynamic parameters such as pulse index (PI), RI, and systolic peak velocity/diastolic peak velocity ratios(S/D), which are suggested to be valuable indicators for endometrial receptivity. While our study provides a promising prospect of HBOT in treating thin endometrium in patients undergoing FET treatments, well-designed prospective studies are needed, and the underlying mechanism remains to be explored. In addition, an optimal time and dose for HBOT need to be investigated.

## Conclusion

In conclusion, this study sheds light on HBOT in patients with resistant thin endometrium during FET treatments. A period of HBOT prior to endometrium transformation helps increase endometrial thickness and facilitate blastocyst implantation; however, the underlying mechanism needs to be explored.

## Data Availability

The datasets used and/or analyzed during the current study are available from the corresponding author on reasonable request.

## Abbreviations

HBOT: hyperbaric oxygen therapy
FET: frozen embryo transfer
PSM: propensity score matching
ET: endometrial thickness
IPR: intrauterine pregnancy rate
IR: embryo implantation rate
BPR: biochemical pregnancy rate
G-CSF: granulocyte colony-stimulating factor
BMI: body mass index.
AFS: the American Fertility Society’s classification of intrauterine adhesions
ATA: atm absolute
HRT: hormone replacement treatment
NC: natural cycle
GnRH-a HRT: GnRH-agonist combined hormone replacement treatment
FSH: follicular stimulating hormone
LH: luteinizing hormone. AMH, anti-Müllerian hormone
IUA: intrauterine adhesion
RI: resistance index
PI: pulse index
S/D: systolic peak velocity/diastolic peak velocity ratios

## References

[1] Check JH, Dietterich C, Check ML, Katz Y (2003). Successful delivery despite conception with a maximal endometrial thickness of 4 mm. Clin Exp Obstet Gynecol.

[2] Gao G, Cui XF, Li S, Ding P, Zhang S, Zhang Y (2020). Endometrial thickness and IVF cycle outcomes: a meta-analysis. Reprod Biomed Online.

[3] Zhang T, Li Z, Ren X, Huang B, Zhu G, Yang W (2018). Endometrial thickness as a predictor of the reproductive outcomes in fresh and frozen embryo transfer cycles: a retrospective cohort study of 1512 IVF cycles with morphologically good-quality blastocyst. Med (Baltim).

[4] Israel R, Isaacs JD, Wells CS, Williams DB, Odem RR, Gast MJ (1996). Endometrial thickness is a valid monitoring parameter in cycles of ovulation induction with menotropins alone. Fertil Steril.

[5] Chen SL, Wu FR, Luo C, Chen X, Shi XY, Zheng HY (2010). Combined analysis of endometrial thickness and pattern in predicting outcome of in vitro fertilization and embryo transfer: a retrospective cohort study. Reprod Biol Endocrinol.

[6] Zhao J, Zhang Q, Wang Y, Li Y (2014). Endometrial pattern, thickness and growth in predicting pregnancy outcome following 3319 IVF cycle. Reprod Biomed Online.

[7] Chen MJ, Yang JH, Peng FH, Chen SU, Ho HN, Yang YS (2006). Extended estrogen administration for women with thin endometrium in frozen-thawed in-vitro fertilization programs. J Assist Reprod Genet.

[8] Feng W, Nie L, Wang X, Yang F, Pan P, Deng X (2021). Effect of oral versus vaginal administration of Estradiol and Dydrogesterone on the proliferative and secretory Transformation of Endometrium in patients with premature ovarian failure and preparing for assisted Reproductive Technology. Drug Des Devel Ther.

[9] Li X, Su Y, Xie QJ, Luan T, Zhang M, Ji X, Liu Y (2021). The Effect of Sildenafil citrate on poor endometrium in patients undergoing frozen-thawed embryo transfer following resection of Intrauterine Adhesions: a retrospective study. Gynecol Obstet Invest.

[10] Tao Y, Wang N (2020). Adjuvant vaginal use of Sildenafil Citrate in a hormone replacement cycle Improved Live Birth Rates among 10,069 women during first frozen embryo transfers. Drug Des Devel Ther.

[11] Xu B, Zhang Q, Hao J, Xu D, Li Y (2015). Two protocols to treat thin endometrium with granulocyte colony-stimulating factor during frozen embryo transfer cycles. Reprod Biomed Online.

[12] Gleicher N, Kim A, Michaeli T, Lee HJ, Shohat-Tal A, Lazzaroni E (2013). A pilot cohort study of granulocyte colony-stimulating factor in the treatment of unresponsive thin endometrium resistant to standard therapies. Hum Reprod.

[13] Chang Y, Peng J, Zhu Y, Sun P, Mai H, Guo Q (2023). How platelet-rich plasma (PRP) intra-uterine injection improve endometrial receptivity of Intrauterine Adhesions in women: a time-series-based self-controlled study. J Reprod Immunol.

[14] Zhang Y, Shi L, Lin X, Zhou F, Xin L, Xu W (2021). Unresponsive thin endometrium caused by Asherman syndrome treated with umbilical cord mesenchymal stem cells on collagen scaffolds: a pilot study. Stem Cell Res Ther.

[15] Zakrzewski W, Dobrzyński M, Szymonowicz M, Rybak Z (2019). Stem cells: past, present, and future. Stem Cell Res Ther.

[16] Sen S, Sen S. Therapeutic effects of hyperbaric oxygen: integrated review. Med Gas Res 2021 Jan-Mar;11(1):30–3. 10.4103/2045-9912.310057 PMC8103971 33642335

[17] Zhou D, Fu D, Yan L, Xie L (2023). The role of hyperbaric oxygen therapy in the treatment of Surgical Site Infections: a narrative review. Med (Kaunas).

[18] Mensah-Kane P, Sumien N (2023). The potential of hyperbaric oxygen as a therapy for neurodegenerative diseases. Geroscience.

[19] Ishihara A (2019). Mild hyperbaric oxygen: mechanisms and effects. J Physiol Sci.

[20] Van Voorhis BJ, Greensmith JE, Dokras A, Sparks AE, Simmons ST, Syrop CH (2005). Hyperbaric oxygen and ovarian follicular stimulation for in vitro fertilization: a pilot study. Fertil Steril.

[21] Mitrović A, Brkić P, Nikolić B, Dragojević S, Zaric O, Ljubić A, Jovanović T (2006). Hyperbaric oxygen and in vitro fertilisation. Aust N Z J Obstet Gynaecol.

[22] Mitrović A, Nikolić B, Dragojević S, Brkić P, Ljubić A, Jovanović T (2006). Hyperbaric oxygenation as a possible therapy of choice for infertility treatment. Bosn J Basic Med Sci.

[23] Leverment J, Turner R, Bowman M, Cooke CJ (2004). Report of the use of hyperbaric oxygen therapy (HBO2) in an unusual case of secondary infertility. Undersea Hyperb Med.

[24] Casper RF (2021). It’s time to pay attention to the Endometrium. Fertil Steril.

[25] Alpha Scientists in Reproductive Medicine and ESHRE Special Interest Group of Embryology. The Istanbul consensus workshop on embryo assessment: proceedings of an expert meeting. Hum Reprod. 2011;26(6):1270–1283. 10.1093/humrep/der037 21502182

[26] Takasaki A, Tamura H, Miwa I, Taketani T, Shimamura K, Sugino N (2010). Endometrial growth and uterine blood flow: a pilot study for improving endometrial thickness in the patients with a thin endometrium. Fertil Steril.

[27] Gardner DK, Lane M, Stevens J, Schlenker T, Schoolcraft WB (2019). Reprint of: blastocyst score affects implantation and pregnancy outcome: towards a single blastocyst transfer. Fertil Steril.

[28] Mahajan N, Sharma S (2016). The endometrium in assisted reproductive technology: how thin is thin?. J Hum Reprod Sci.

[29] Kasius A, Smit JG, Torrance HL, Eijkemans MJ, Mol BW, Opmeer BC, Broekmans FJ (2014). Endometrial thickness and pregnancy rates after IVF: a systematic review and meta-analysis. Hum Reprod Update.

[30] Ribeiro VC, Santos-Ribeiro S, De Munck N, Drakopoulos P, Polyzos NP, Schutyser V, Verheyen G, Tournaye H, Blockeel C (2018). Should we continue to measure endometrial thickness in modern-day medicine? The effect on live birth rates and birth weight. Reprod Biomed Online.

[31] Dain L, Bider D, Levron J, Zinchenko V, Westler S, Dirnfeld M (2013). Thin endometrium in donor oocyte recipients: enigma or obstacle for implantation?. Fertil Steril.

[32] Miwa I, Tamura H, Takasaki A, Yamagata Y, Shimamura K, Sugino N (2009). Pathophysiologic features of “thin” endometrium. Fertil Steril.

[33] Sundström P (1998). Establishment of a successful pregnancy following in-vitro fertilization with an endometrial thickness of no more than 4 mm. Hum Reprod.

[34] El-Toukhy T, Coomarasamy A, Khairy M, Sunkara K, Seed P, Khalaf Y, Braude P (2008). The relationship between endometrial thickness and outcome of medicated frozen embryo replacement cycles. Fertil Steril.

[35] Glujovsky D, Pesce R, Sueldo C, Quinteiro Retamar AM, Hart RJ, Ciapponi A (2020). Endometrial preparation for women undergoing embryo transfer with frozen embryos or embryos derived from donor oocytes. Cochrane Database Syst Rev.

[36] Catt JW, Henman M (2000). Toxic Effects of Oxygen on human embryo development. Hum Reprod.

[37] Schoots MH, Gordijn SJ, Scherjon SA, van Goor H, Hillebrands JL (2018). Oxidative stress in placental pathology. Placenta.

[38] Jain KK. Physical, physiological, and biochemical aspects of hyperbaric oxygenation. Textbook of Hyperbaric Medicine. 2017; Switzerland AG Springer Nature:11–22.

[39] Lindenmann J, Kamolz L, Graier W, Smolle J, Smolle-Juettner FM (2022). Hyperbaric oxygen therapy and tissue regeneration: a Literature Survey. Biomedicines.

[40] Fosen KM, Thom SR (2014). Hyperbaric oxygen, vasculogenic stem cells, and wound healing. Antioxid Redox Signal.

[41] Yuan J, Handy RD, Moody AJ, Bryson P (2009). Response of blood vessels in vitro to hyperbaric oxygen (HBO): modulation of VEGF and NO(x) release by external lactate or arginine. Biochim Biophys Acta.

[42] Sheikh AY, Gibson JJ, Rollins MD, Hopf HW, Hussain Z, Hunt TK (2000). Effect of hyperoxia on vascular endothelial growth factor levels in a wound model. Arch Surg.

[43] Liu X, Ye N, Liu S, Guan J, Deng Q, Zhang Z, Xiao C, Ding Z, Zhang B, Chen X (2021). Hyperbaric oxygen boosts PD-1 antibody delivery and T cell infiltration for augmented Immune responses against solid tumors. Adv Sci (Weinh).

[44] Li K, Gong Y, Qiu D, Tang H, Zhang J, Yuan Z, Huang Y, Qin Y, Ye L, Yang Y (2022). Hyperbaric oxygen facilitates teniposide-induced cGAS-STING activation to enhance the antitumor efficacy of PD-1 antibody in HCC. J Immunother Cancer.

[45] Chen XX, You JH, Ma H, Zhou M, Huang C (2023). Efficacy and safety of hyperbaric oxygen therapy for fibromyalgia: a systematic review and meta-analysis. BMJ Open.

[46] Kitala D, Łabuś W, Kozielski J, Strzelec P, Nowak M, Knefel G (2022). Preliminary Research on the Effect of Hyperbaric Oxygen Therapy in patients with Post-COVID-19 syndrome. J Clin Med.

[47] Velure GK, Müller B, Hauken MA (2022). Symptom burden and health-related quality of life six months after hyperbaric oxygen therapy in cancer survivors with pelvic radiation injuries. Support Care Cancer.

[48] Ben K, Reut, Michal Y, Tamar RMA, Sarit A, Itai G, Lilach MH, Ariel H, Alon K (2020). Fertility patients under COVID-19: attitudes, perceptions and psychological reactions. Hum Reprod.

